# Questions and Certainty in Diagnosis and Management of Acute Type B Aortic Dissection

**DOI:** 10.31083/RCM26807

**Published:** 2025-02-20

**Authors:** Luigi Lovato, Maria Adriana Cocozza, Alessandro Onori, Rossella Fattori

**Affiliations:** ^1^Pediatric and Adult Cardiothoracic and Vascular, Oncohematologic and Emergency Radiology Unit, IRCCS Azienda Ospedaliero-Universitaria di Bologna, 40138 Bologna, Italy; ^2^Department of Radiological Sciences, Oncology and Pathology, I.C.O.T. Hospital, Sapienza University of Rome, 04100 Latina, Italy; ^3^Department of Experimental, Diagnostic and Specialty Medicine (DIMES), University of Bologna, 40138 Bologna Italy

**Keywords:** type B aortic dissection (TBAD), thoracic endovascular aortic repair (TEVAR), computed tomography angiography (CTA)

## Abstract

Type B aortic dissection (TBAD) is a severe cardiovascular condition that requires timely diagnosis and intervention to prevent life-threatening complications. The aim of this review was to focus on the most crucial and controversial aspects of contemporary TBAD management. It is recognized that in the acute phase, computed tomography angiography (CTA) plays an essential role in evaluating the extent of the dissection and monitoring disease progression. CTA has significantly improved the management of TBAD by providing detailed assessments of aortic anatomy and dynamic flow changes, positioning it as the cornerstone imaging modality for identifying acute high-risk patients who may require early intervention. Recently, new advances in magnetic resonance imaging (MRI) and positron emission tomography (PET) technology have the potential to provide further information beyond imaging alone. However, such sophisticated techniques should be reserved for stable patients after the acute phase. After decades of medical therapy and high risk surgery, thoracic endovascular aortic repair (TEVAR) has emerged as a minimally invasive alternative to open surgery for complicated TBAD, offering lower perioperative morbidity and mortality. Nevertheless, its use in uncomplicated TBAD remains a topic of ongoing debate. While recent studies suggest that preemptive TEVAR combined with optimal medical therapy may reduce late adverse events and improve long-term outcomes, these findings remain controversial. This review critically analyzes the current literature on both diagnosis and TEVAR treatment, evaluating these controversies in the context of clinical practice.

## 1. Introduction

Type B aortic dissection (TBAD) is a severe and life-threatening cardiovascular condition characterized by a tear in the inner layer of the aorta, specifically occurring in the descending thoracic aorta. This pathology necessitates a prompt, detailed diagnosis, and subsequent appropriate management to prevent fatal complications. Understanding and knowledge of TBAD has evolved over the years. Initially based on medical therapy as the gold standard for uncomplicated TBAD, in the past few years, thoracic endovascular aortic repair (TEVAR) emerged for complicated TBAD as a minimally invasive alternative to open surgical repair, showing reduced perioperative morbidity and mortality rates, which become similar to those achieved in uncomplicated TBAD. However, the difficult identification of unstable patients and the decision to proceed with TEVAR is still a matter of debate that must be carefully weighted at patient clinical presentation [1, 2, 3].

This review aims to provide an overview of the current state of diagnosis and management of TBAD, focusing on the latest imaging modalities because of their potential role in the diagnostic pathway, especially including the identification of high-risk features that may influence the process of decision-making.

## 2. Definition

According to the traditional Stanford classification, the most commonly used, TBADs are identified as any dissection where the tear originates beyond the left subclavian artery, although this classification does not specify the distal extent of the dissection [4].

The recent reporting standards from the Society of Thoracic Surgery (STS) and the Society of Vascular Surgery (SVS) try to offer a more consistent and meaningful framework for characterizing TBAD. A significant addition to these standards is the inclusion of dissection tears that originate within the aortic arch. Now, TBAD is defined as any aortic dissection with an entry tear originating in aortic zone 1 or further distal. Additionally, TBAD is characterized by two subscripts that indicate the proximal and distal extent of the dissection [5].

### 2.1 Definition of Acute, Subacute and Chronic Type B Aortic Dissection

The classification of the phases of aortic dissection has been refined by the European interdisciplinary consensus, which categorizes type B dissections based on their spontaneous mortality rates and temporal progression [2].

According to these recommendations, a type B dissection is defined as “acute” if it occurs within the first two weeks of symptom onset, “subacute” if it occurs between two and six weeks, and “chronic” if it persists beyond six weeks. Additionally, the International Registry of Acute Aortic Dissection (IRAD) investigators have proposed a more detailed temporal classification to further delineate survival outcomes: “hyperacute” (within 24 hours), “acute” (2–7 days), “subacute” (8–30 days), and “chronic” (beyond 30 days) [6].

Survival rates tend to decrease progressively across these phases, with cumulative survival rates ranging from 94–99% in the hyper acute phase, 82–93% in the acute phase, 77–92% in the subacute phase, and 73–91% in the chronic phase, irrespective of the treatment modality [6, 7].

This important early and long term mortality highlights the importance of timely and appropriate intervention. Continuous surveillance imaging is crucial for all types of TBAD, as it provides essential information for monitoring the condition’s progression and guiding management decisions, regardless of the time since the onset of symptoms or the specific risk stratification.

### 2.2 Definition of Complicated and Uncomplicated Type B Aortic Dissection

There is a lack of uniform criteria for defining complicated TBAD which typically should necessitate a more aggressive approach. Traditionally, complicated TBAD is characterized by the presence of signs of hemodynamic instability such as malperfusion syndrome, refractory hypertension, or persistent intractable pain. It is also important to recognize signs of impending rupture such as periaortic hematoma or persistent thoracic pain, or significant aneurysmal expansion in the short term, documented by imaging modalities. However, some of these apparently simple clinical signs may be difficult to identify. In contrast, uncomplicated TBAD presents without these clinical manifestations and is usually managed with optimal medical therapy (OMT) targeting blood pressure control and close monitoring. However, there is a growing interest in the use of TEVAR even in this category, with the approach gaining traction within the scientific community. This shift is a topic of active debate, as the potential benefits of TEVAR in uncomplicated TBAD are being increasingly explored and discussed [8].

The literature suggests that approximately 25% of patients presenting with acute TBAD experience complications at admission, which significantly increases the risk of early mortality if not promptly addressed [9, 10, 11]. Data from the International IRAD registry indicate that in-hospital mortality is significantly higher in patients with TBAD who experience refractory hypertension or pain compared to those without these complications (35.6% vs. 1.5%) [10, 11].

The expert panel consensus, by Fattori *et al*. [2], recommends defining complicated TBAD by the presence of organ malperfusion, particularly when confirmed by laboratory markers and imaging data. Persistent hypertension should only be considered a complication if it remains uncontrolled despite full medical therapy because it may imply a subclinical renal malperfusion. Lastly, the detection of increasing peri-aortic hematoma or hemorrhagic pleural effusion on serial imaging should prompt concern for imminent rupture. As research advances, the distinction between complicated and uncomplicated TBAD continues to evolve, emphasizing the need for personalized management strategies that consider individual patient risk factors and imaging findings [9].

**Fig. 1.**
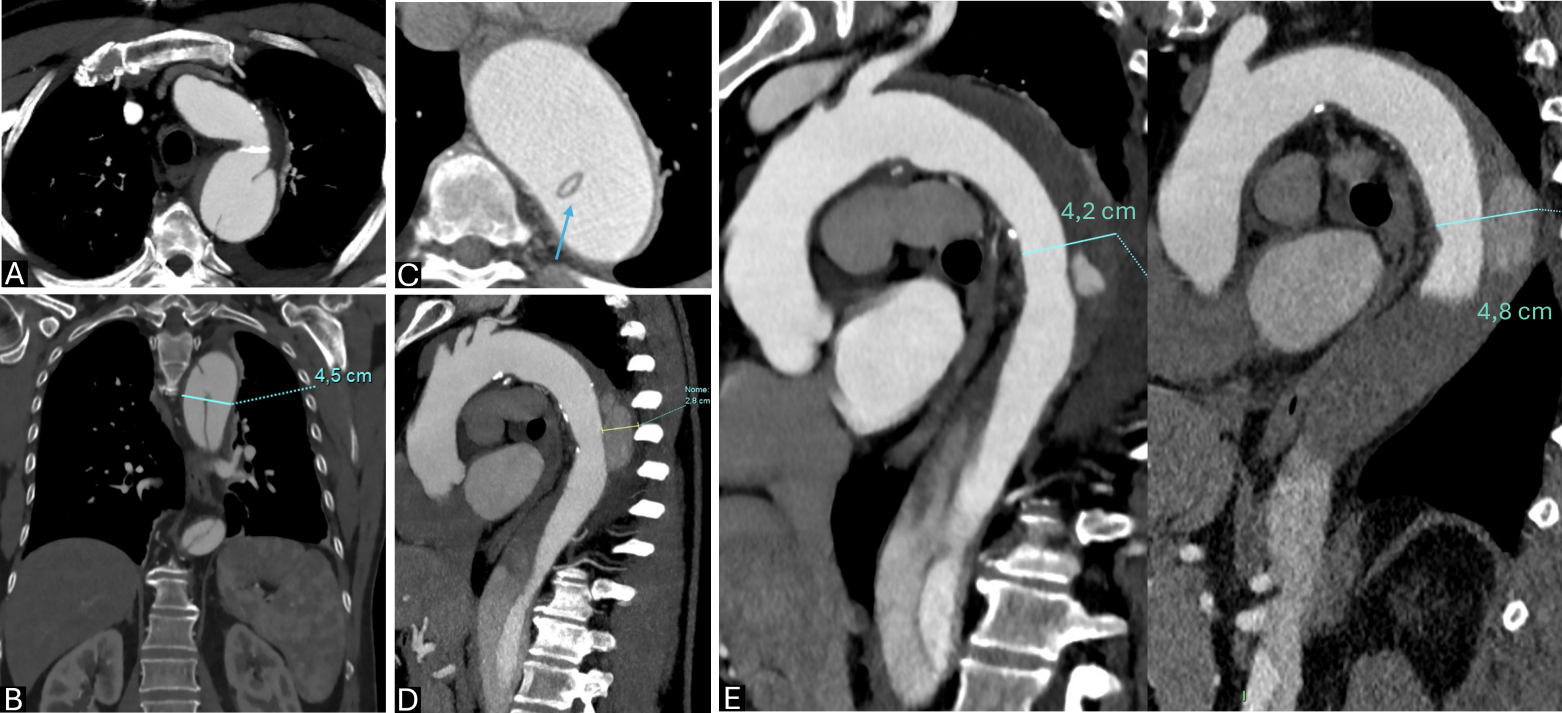
**Type B aortic dissection (TBAD) high-risk features at computed tomography angiography (CTA)**. (A) Wide entry tear (>1 cm); (B) aortic dimensions >40 mm at first assessment; (C) circumferential intimal flap (arrow); (D) false lumen (FL) partial thrombosis and large dimensions (>22 mm); (E) descending aorta enlargement (>5 mm) at serial imaging (15 days follow-up CTA).

Signs of disease progression in TBAD are critical to define, as they can indicate worsening of the condition and the need for intervention. Several risk factors have been identified that contribute to the progression of TBAD from an uncomplicated to a complicated state (Fig. 1). These key signs are summarized in Table 1 (Ref. [10, 11, 12, 13, 14, 15, 16, 17, 18, 19, 20, 21, 22, 23, 24]) A key predictor is the size of the aorta and false lumen (FL), an aortic diameter greater than 40 mm or a FL diameter exceeding 22 mm significantly increases the risk of complications. Additional anatomical factors include the presence of a large proximal entry tear and persistent FL patency, which can lead to continued aortic expansion and potential rupture [25, 26].

**Table 1.** **High-risk imaging features of TBAD**.

Aortic diameters and features
Baseline ≥40 mm [12, 13, 14]
Length of the ascending aorta ≤92 mm [15]
Tortuosity ≥1.17 [11]
FL characteristics
Partially thrombosed FL [16]
Diameter ≥22 mm [17]
Area ≥922 mm^2^ [18, 19]
TL/(TL+FL) <25% [20]
TLV/FLV <0.8 [21]
FL located at inner (vs outer) aortic curvature [22]
Elliptic (vs circular) TL and saccular FL configuration [22]
FL circumferential extent ≥249° [23]
Decreased FL outflow (mL/min) [23]
Entry tear
Single ET (vs multiple re-entry tears) [24]
Primary ET diameter ≥10 mm [10]
Close ET proximity to LSCA [10]
Intramural Hematoma [15]
Abdominal vessel involvement
Visceral malperfusion [11]

FL, false lumen; TL, true lumen; FLV, false lumen volume; TLV, true lumen volume; ET, entry tear; LSCA, left subclavian artery; TBAD, type B aortic dissection.

The study by Miyoshi *et al*. [27] found that moderate aortic enlargement (2 to <5 mm) within two weeks is a significant risk factor for aorta-related adverse events in patients with uncomplicated type B acute aortic syndrome. Specifically, these patients had significantly lower aorta-related event-free survival rates compared to those without such enlargement, indicating a need for careful follow-up and potential endovascular treatment.

From a clinical perspective, persistent or recurrent pain, especially in the chest or back, is often a warning sign of disease progression. This pain may indicate ongoing dissection, impending rupture, or complications like malperfusion syndromes. Systemic signs such as fever might suggest infection or inflammation, while neurological deficits, including symptoms like weakness or paralysis, could point to spinal cord ischemia, especially in cases where malperfusion affects critical blood flow to the spine [28].

Computed tomography angiography (CTA) plays a central role in identifying signs of disease progression. Early detection of impending rupture or dissection extension is critical, with markers such as rapid aortic expansion, the presence of periaortic hematoma, contrast extravasation and the new appearance of pleural or pericardial effusion, all of which suggest a compromised aortic structure. One important radiological feature is the “beak sign”, an acute angle formed by the dissection flap, which signifies a severe pressure differential and increased rupture risk. Additionally, CTA is essential for diagnosing malperfusion, characterized by the narrowing or occlusion of branch vessels, reduced organ enhancement or ischemic hypodensity in affected tissues [29].

Early identification of these risk factors through advanced imaging techniques and close clinical monitoring is vital, as it allows for timely intervention that can prevent progression to complicated TBAD and improve overall patient outcomes.

## 3. Literature Review

The literature search was conducted on computerized databases, including PubMed, Ovid Medline, and the Cochrane Library, including studies from January 2006 to February 2024. Search strings included “type B aortic dissection” combined with the term “endovascular treatment” or “TEVAR”. Subheadings for the search were the terms “acute”, and “complicated” and “uncomplicated”.

The search was limited to studies on humans and adults only, with at least an abstract available in English.

After potentially relevant studies were identified, additional tangential searches were conducted using related study links within PubMed or within a reference list of published papers.

For the analysis, studies evaluating TEVAR management of acute type B dissection were eligible if they included at least 15 patients with type B aortic dissection and reported at least 1 clinically relevant outcome.

In evaluating multiple publications of overlapping patient populations, studies were evaluated by the center(s) and patient enrollment dates, and the most recent and/or most complete series was selected to extract as many relevant outcomes as possible.

To define the timing of type B aortic dissection, acute presentation had to be within 14 days of onset of symptoms. To define the complicated presentation, aortic rupture, visceral malperfusion, limb ischemia, refractory pain/hypertension despite adequate medical treatment had to be present.

Studies dealing with less than 15 patients, with uncomplicated type B aortic dissection or type A aortic dissection were excluded.

The major endpoint was early (in-hospital and 30-day) mortality. Secondary endpoints included early (in-hospital/30 days) stroke and spinal cord ischemia, FL retraction, endoleaks, reintervention rate, long-term survival and, if available, aortic event-free survival.

An ancillary analysis was conducted to investigate radiological high-risk stigmata as outcome predictors of complicated type B aortic dissection on CTA.

The analysis of TEVAR case series for acute complicated type B aortic dissection reveals significant variability in early mortality, major complications, and long-term outcomes across different studies (Table 2, Ref. [30, 31, 32, 33, 34, 35, 36, 37, 38, 39, 40, 41, 42, 43, 44, 45, 46, 47, 48, 49, 50, 51, 52, 53, 54, 55, 56, 57, 58, 59, 60, 61, 62, 63]).

**Table 2.** **Result summary for TEVAR case series in acute complicated TBAD**.

Author and Year	Study type	n	Early mortality	SCI	CVA	FL retraction	EL	2nd procedure	Mean follow-up (months)	Survival rate (%)	Aortic event freedom rate (%)
			n (%)	n (%)	n (%)	n (%)	n (%)	n (%)			
Böckler 2009 [30]	R	23	6 (26)	0	0	11 (44)	2 (5)	3 (13.5)	24 (0–56)	1 yr: 62	1 yr: 64
										5 yrs: 62	5 yrs: 45
Chen 2006 [31]	P	23	1 (4.3)	0	1 (4.3)	NA	1 (4.3)	NA	27.5 ± 14.2	NA	NA
Steingruber 2008 [32]	R	38	9 (23.7)	0	1 (2.6)	2 (5.7)	9 (23.7)	4 (10.5)	33	1 yr: 81.5	NA
										5 yrs: 69	
Steingruber 2008 [32]	R	35	3 (8.5)	0	0	21 (59)	8 (22)	2 (5.7)	34	5 yrs: 78.4	NA
Rodriguez 2008 [33]	R	59	1 (1.7)	3 (5.1)	3 (5.1)	22 (64.7)	7 (6.6)	2 (3)	15.6	NA	NA
Sayer 2008 [34]	R	38	1 (2.6)	0	2 (5.3)	27 (70)	5 (13)	8 (21)	30	93	55
Alves 2009 [35]	R	45	3 (6.7)	1 (2)	5 (11)	34 (75.5)	11 (24)	4 (8.8)	35.9 ± 28.5	35.9 months: 78	35.9 months: 79
Conrad 2009 [36]	R	33	4 (12)	2 (6)	4 (12)	NA	3 (9)	NA	12	1 yr: 75	NA
Feezor 2009 [37]	P	33	7 (21.2)	5 (15.1)	4 (12.1)	NA	7 (35)	16 (48)	5	NA	NA
Khoynezhad 2009 [38]	R	41	4 (9.8)	0	5 (12.2)	22 (85)	5/28 (18)	2/28 (7)	On 28 pts 36 ± 27 (1–88)	On 28 pts	NA
Kim 2011 [39]										1 yr: 82	
										5 yrs: 78	
Manning 2009 [40]	R	45	5 (11.1)	4 (8.9)	2 (4.4)	Type IIIb 32%	NA	22% of IIB	30 ± 9 for IIIa	NA	NA
									29 ± 18 for IIIb		
Sze 2009 [41]	R	23	4 (17.4)	1 (4.3)	2 (8.7)	NA	11 (48)	NA	22.3 (0–92)	NA	NA
Botsios 2010 [42]	R	32	3 (9.3)	1 (3.1)	NA	10 (31)	2 (6)	3 (9)	32 ± 18.7 (2–60)	NA	NA
Ehrlich 2010 [43]	R	32	4 (12.5)	3 (9.4)	NA	NA	2 (6)	15 (46)	26 ± 23	1 yr: 81	1 yr: 78
										5 yrs: 76	5 yrs: 61
Parsa 2010 [44]	R	22	1 (4.5)	1 (4.5)	0	8 (35)	6 (27)	1 (5)	7.1 (1–38)	NA	38 months: 94
Zeeshan 2010 [45]	R	45	2 (4.4)	6 (13.3)	3 (6.7)	30 (77)	1 (2)	7 (15.5)	37	1 yr: 82	5 yrs: 89
										5 yrs: 79	
Tang 2011 [46]	R	30	1 (3.3)	0	NA	6 (20)	1 (3.3)	0	12 ± 8 (1–19)	NA	NA
O’Donnell 2011 [47]	R	28	2 (7.2)	1 (3.6)	3 (10.7)	8/10 (80)	NA	2 (7.1)	21 (1–45)	NA	NA
Shu 2011 [48]	R	45	2 (4.4)	0	0	25 (55.5)	3 (6.6)	1 (2)	13 (1–36)	1 yr: 95.6	NA
										3 yrs: 96.6	
Hanna 2014 [49]	R	50	0	0	0	NA	5 (10)	13 (26)	33.8 (12.3–56.6)	5 yrs: 84	NA
Wiedemann 2014 [50]	R	110	13 (12)	5 (4.5)	0	8 (7.3)	9 (8)	11 (10.9)	37 (0–144)	1 yr: 85	NA
										5 yrs: 73	
Afifi 2015 [51]	R	37	5 (13.5)	2 (5.4)	4 (10.8)	NA	1 (2.7)	4 (10.8)	55.2 (22.8–93.6)	1 yr: 78.4	NA
										5 yrs: 58.8	
Väärämäki 2016 [52]	P	16	1 (6.3)	1 (6.3)	4 (25)	NA	6 (37.5)	NA	55 (1–160)	1 yr: 85	NA
										3 yrs: 78	
										5 yrs: 61	
Lou 2018 [53]	R	80	4 (5)	2 (2.5)	6 (7.5)	NA	NA	24 (30)	12	1 yr: 84.1	NA
Chou 2018 [54]	R	26	2 (7.7)	1 (3.8)	0	8 (33.3)	4 (15)	2 (7.7)	27.5 ± 19.1	3 yrs: 77.6	NA
Clough 2019 [55]	R	64	1 (1.5)	NA	NA	17 (27.3)	NA	NA	51 (1–132)	1 yr: 94	1 yr: 75.6
										5 yrs: 74.8	5 yrs: 58.7
Pruitt 2020 [56]	R	159	21 (13)	10 (7)	12 (8)	NA	59 (37)	30 (20)	16 ± 15	1 yr: 75	1 yr: 78 ± 7
										5 yrs: 73	2 yr: 73 ± 9
Li 2020 [57]	R	165	5 (3)	0	0	NA	16 (9.7)	19 (12.1)	68.1 ± 22.9	1 yr: 88.4	NA
										5 yrs: 58.7	
Lou 2020 [58]	R	91	6 (5)	3 (3)	6 (5)	44 (54)	1 (1)	9 (10)	37.2 (14.4–58.8)	1 yr: 92	1 yr: 89.2
										5 yrs: 85	5 yrs: 76.4
Jin 2021 [59]	R	63	2 (3.1)	0	0	57 (91)	1 (1.6)	1 (1.6)	30.1 ± 18.9	1 yr: 85	NA
Hong 2021 [60]	R	18	0	0	4 (22.2)	15 (83.3)	2 (11.1)	2 (11.1)	34.5 (12–80)	1 yr: 100	NA
Herajärvi 2022 [61]	R	59	7 (11.9)	2 (3.3)	4 (6.8)	NA	NA	14 (23.7)	58.8 ± 45.6	1 yr: 83	NA
										5 yrs: 60	
										10 yrs: 42	
Spinelli 2023 [62]	R	102	3 (2.9)	3 (2.9)	2 (2.0)	NA	NA	7 (6.9)	24 (9.6–33.6)	1 yr: 88.8	NA
										3 yrs: 79.1	
Rossi 2024 [63]	P	56	1 (1.8)	5 (8.9)	1 (1.8)	56 (100)	1 (1.8)	2 (3.6)	12	1 yr: 87.5	NA
TOTAL		1776	134 (7.5)	62 (3.5)	72 (4)	445 (NA)	183 (NA)	208 (NA)	NA	NA	NA

R, retrospective study; P, prospective study; SCI, spinal cord ischemia; CVA, cerebrovascular accident; FL, false lumen; EL, endoleak; NA, not applicable; TEVAR, thoracic endovascular aortic repair; TBAD, type B aortic dissection; yr, year; pts, patients.

The early mortality rates in the reviewed studies ranged from 0% to 26%, with an overall mortality of 7.5% (134 out of 1776 patients). This wide variability can be attributed to differences in study design (prospective vs. retrospective), patient selection criteria, and procedural expertise across centers. Böckler *et al*. [30] reported the highest early mortality (26%), which may reflect more severe cases or differences in management, while several studies, such as Hanna *et al*. [49], reported 0% early mortality, suggesting potential advances in perioperative care or patient selection in more recent studies.

Complications such as spinal cord ischemia (SCI) and cerebrovascular accidents (CVA) are critical concerns in the management of TBAD. The overall incidence of SCI was 3.5% (62 patients), with the highest rate reported by Feezor *et al*. [37] at 15.1%. The incidence of CVA was slightly higher, at 4% overall (72 patients), with a maximum of 12.2% reported by Khoynezhad *et al*. [38]. These findings underscore the importance of careful perioperative monitoring and strategies aimed at reducing neurological complications, such as cerebrospinal fluid drainage and optimized blood pressure control.

A key indicator of procedural success in TEVAR is the reduction or retraction of the FL and the prevention of endoleaks (ELs). Table 2 shows that FL retraction was not universally reported, but in studies where it was assessed, rates were high. For example, Khoynezhad *et al*. [38] reported FL retraction in 85% of patients, and Jin *et al*. [59] achieved a retraction rate of 91%. However, endoleaks were still observed in 10.3% (183 patients), suggesting that while TEVAR is effective in promoting FL thrombosis, ongoing surveillance for endoleaks is critical for long-term success.

The need for reinterventions is another critical aspect of TEVAR outcomes. The overall rate of second procedures was 11.7% (208 patients), with the highest rate of 48% reported by Feezor *et al*. [37]. These findings highlight that a significant proportion of patients may require further interventions, either for persistent endoleaks or progression of the dissection, suggesting the need for long-term follow-up and consideration of adjunctive therapies in certain cases.

Long-term survival data were inconsistently reported across studies, but where available, 1-year survival ranged from 62% to 100%, with 5-year survival varying from 45% to 85%. The freedom from aortic events also showed variability, with some studies reporting as low as 45% at 5 years [30] and others achieving rates as high as 89% [45]. This underscores the heterogeneity of patient outcomes post-TEVAR and highlights the need for individualized treatment strategies and vigilant long-term surveillance. In analyzing the data from older versus more recent studies in the TEVAR case series for acute complicated type B aortic dissection, there appears to be an observable trend in outcomes, especially regarding early mortality, major complications, and long-term survival. Older studies, such as those from 2006 to 2010, reported higher early mortality (up to 26%), more frequent neurological complications like spinal cord ischemia (up to 15.1%) and higher reintervention rates (up to 48%). In contrast, more recent studies from 2021 onward show significantly lower early mortality (as low as 1.8%), reduced complications (SCI rates below 9%), and fewer reinterventions, with improved long-term survival rates reaching 85% at five years.

These trends demonstrate substantial improvements in patient outcomes over time, driven by advancements in technology, procedural techniques and perioperative care. Despite these advances, the variability in outcomes underscores the importance of individualized treatment strategies, careful patient selection, and long-term follow-up to optimize success and reduce the risks of complications such as endoleaks and the need for reintervention. As TEVAR continues to evolve, ongoing refinement in both the technique and postoperative management will be essential to further enhance outcomes in patients with acute complicated TBAD.

## 4. Guidelines and Controversies 

The management of TBAD involves complex decision-making, particularly when distinguishing between complicated and uncomplicated cases. Current guidelines recommend medical therapy as the first-line treatment for uncomplicated TBAD, with the goal of controlling hypertension and reducing stress on the aortic wall [3, 5].

However, the role of TEVAR in uncomplicated TBAD remains a topic of ongoing debate. While TEVAR is well-established for treating complicated TBAD due to its minimally invasive nature and lower morbidity compared to open surgery, its use in uncomplicated cases is more controversial with current guidelines lacking specificity on the best therapeutic window [64].

This controversy primarily centers on the timing and necessity of preemptive TEVAR in uncomplicated TBAD. Some recent studies suggest that when TEVAR is combined with optimal medical therapy, it can significantly reduce late adverse events and mortality, despite a higher initial stroke risk [65, 66].

Iannuzzi *et al*. [67] reported that TEVAR was associated with a 32% reduction in mortality risk compared to medical therapy alone, with five-year survival rates of 75.9% for TEVAR versus 59.8% for medical therapy. These findings are in contrast to earlier results from the INSTEAD trial, which did not show a survival benefit of TEVAR at one year but did observe improved aorta-specific survival at five years. These differing outcomes have fueled an ongoing debate about the appropriate timing and patient selection for TEVAR in uncomplicated TBAD, raising important questions about the potential risks, costs, and long-term consequences of its broader application [67].

Another significant point of contention is the timing of TEVAR. Evidence suggests that performing TEVAR during the subacute phase of TBAD, rather than the acute phase, may result in better outcomes, including improved aortic remodeling and fewer complications such as retrograde type A dissection (RTAD) and aortic rupture [68]. While TEVAR is beneficial, it is important to recognize that it also carries the risk of severe complications, particularly RTAD, which, although rare, presents a high mortality rate [69, 70]. RTAD incidence ranges from 2.5% to 10%, with most cases occurring within the first year and nearly 40% within the first 30 days post-procedure [71, 72].

Key risk factors for RTAD include proximal landing zones located near the left subclavian artery, manipulation of the aortic arch, stent oversizing and ballooning during deployment. The risk of developing RTAD is further heightened in patients with large ascending aortas (≥4 cm) or those with partially or fully thrombosed false lumens [73].

To reduce the risk of RTAD, device designs have evolved to minimize the presence of bare-metal struts at the proximal attachment zones, although the impact of these struts remains a topic of debate. The high mortality rate and unpredictability associated with RTAD have significantly influenced stent design and procedural strategies, highlighting the necessity for precision and vigilant monitoring throughout the intervention process [73, 74]. Prompt recognition and intervention are crucial for managing RTAD due to its significant risks. Immediate surgical intervention is often necessary to mitigate complications and enhance patient outcomes. The preferred treatment typically involves reoperation on the aortic arch, which may include arch replacement or *in situ* repair with a tailored graft. In some cases, a hybrid approach that combines open surgery and endovascular techniques may be employed, depending on the patient’s anatomy, the extent of the dissection, and any comorbidities. In cases where the dissection tear is very proximal, especially in Zone 2, and accompanied by a moderate periaortic hematoma, a more aggressive treatment approach may be required. Specialized centers might utilize the “frozen elephant trunk” (FET) procedure for secure proximal repair [75, 76].

Meta-analyses have supported the approach of performing TEVAR in the subacute phase, indicating that patients treated with TEVAR in the subacute phase experience lower perioperative complications and mortality, likely because the increased fragility of the dissection flap during the acute phase heightens the risk of procedural complications [77].

Despite TEVAR’s potential to improve long-term aortic remodeling, the optimal timing for its application remains unresolved, with current guidelines lacking specificity on the best therapeutic window.

The analysis of various registries and trials on TEVAR for acute complicated type B aortic dissection reveals significant variability in early mortality, complications and long-term outcomes, as evidenced in Table 3 (Ref. [9, 10, 19, 78, 79, 80, 81, 82, 83, 84, 85, 86, 87]).

**Table 3.** **Result summary for TEVAR registries and trials in acute complicated type B aortic dissection**.

First author and Year	Registry/trial	n	Early mortality	SCI	CVA	FL retraction	EL	2nd procedure	Mean follow-up (months)	Survival rate (%)	Aortic event freedom rate (%)
			n (%)	n (%)	n (%)	n (%)	n (%)	n (%)			
Fattori 2008 [9], Tsai 2006 [10]	IRAD	43	5 (11.6)	1 (2.3)	2 (3.4)	NA	NA	NA	27.6	On 29 pts	NA
										1 yr: 88.9	
										3 yrs: 76.2	
Torsello 2010 [78]	TRAVIATA	32	0	0	0	14 (43.6)	6 (6.5)	3 (9.3)	23.1 ± 10.1	1 yr: 95.5	NA
										2 yrs: 87.4	
										3 yrs: 76.4	
Riambau 2011 [79]	RESTORE	25	5 (20)	5 (20)	2 (2)	NA	2 (7)	NA	10.1 ± 12	2 yrs: 84	NA
Lombardi 2014 [80]	STABLE I	55	3 (5.5)	1 (1.8)	6 (10.9)	48 months:	NA	4 (10)	24	1 yr: 90	2 yrs: 88.9
						15/31 (48.4)				2 yrs: 87.1	
Ehrlich 2013 [81]	TALENT	29	5 (17.2)	0	3 (10)	18 (62)	4 (13.7)	1 (3.4)	53 ± 41	1 yr: 79	1 yr: 82
										5 yrs: 61	5 yrs: 77
Heijmen 2014 [82]	VIRTUE	50	4 (8)	1 (2.0)	4 (8)	20 (39)	0	4 (8)	36	3 yrs: 73	20
Lin 2021 [83]	RCT	42	5 (11.9)	0	1 (2)	20 (47.6)	3 (7.1)	2 (4.7)	60	2 yrs: 84.7	NA
Bavaria 2022 [84]	NRCT	50	5 (10)	0	0	16/18 (89)	5 (10)	7 (14)	60	65	83
Lombardi 2022 [85]	STABLE II	73	5 (6.8)	5 (6.8)	5 (6.8)	15 (20.7)	NA	6 (8.2)	60	5 yrs: 68.9	NA
Nienaber 2009, 2013 [19, 86]	INSTEAD	70 (acute and chronic uncomplicated)	2 (2.8)	2 (2.8)	1 (1.5)	5 yrs:	NA	13 (18.5)	60	2 yrs: 88.9 ± 3.7	2 yrs: 94.4 ± 2.7
	INSTEAD-XL					48/53 (90.6)				5 yrs: 88.9 ± 3.7	5 yrs: 93.1
Brunkwall 2014 [87]	ADSORB	30 (uncomplicated)	0	0	0	13 (43)	NA	NA	12	1 yr: 100	57

RCT, randomized controlled trial; NRCT, non-randomized prospective controlled trial; SCI, spinal cord ischemia; CVA, cerebrovascular accident; FL, false lumen; EL, endoleak; NA, not applicable; TEVAR, thoracic endovascular aortic repair; yr, year; pts, patients.

Clinical trials such as INSTEAD and ADSORB, which sought to evaluate the benefits of TEVAR in Type B aortic dissections, have revealed significant biases and limitations that complicate the establishment of clear treatment protocols [86, 87].

The INSTEAD trial suggested that stent-grafts might offer long-term benefits, but it did not show a significant reduction in mortality at two years. Mortality in the INSTEAD trial at two years was similar between groups, with a 10.2% mortality rate in the TEVAR group and 11.1% in the Best Medical Therapy (BMT) group. However, the extended follow-up in the INSTEAD-XL trial demonstrated that TEVAR provided substantial long-term advantages, with improved aortic stability and a significant reduction in fatalities at five years compared to BMT alone. At five years, the mortality rate in the TEVAR group was 11.1%, compared to 19.3% in the BMT group [79]. Despite the absence of early mortality benefits, the findings underscore TEVAR’s role in preventing long-term complications such as FL expansion (observed in 20% of the BMT group) and aortic rupture, highlighting its value beyond the initial two-year window.

The ADSORB trial, aimed at assessing the efficacy of stent-grafting in the early stages of uncomplicated dissection, has been criticized for design flaws, including the inclusion of very young patients who may have underlying connective tissue disorders and the failure to account for advancements in stent-graft technology and medical therapy over the past decade. The VIRTUE Registry provides additional insights, particularly regarding the timing and outcomes of TEVAR across acute, subacute, and chronic phases of TBAD [82].

It found that the aorta retains its capacity for remodeling beyond the traditional two-week window post-symptom onset, especially in subacute dissections. This suggests that TEVAR may still be beneficial in this phase, offering low dissection-related mortality at three years post-procedure. However, the study also highlighted a relatively high rate of reinterventions, particularly in chronic dissections, suggesting that more extensive initial treatment may be necessary in these cases.

The existing trials are limited by several factors that impact their clinical relevance. Many rely on retrospective data with small patient populations, which introduces bias and reduces the statistical power and reliability of the results. Additionally, many studies are mono-device, assessing outcomes with only a single type of stent-graft, which restricts the generalizability of their findings across different devices and broader clinical applications. Another key limitation is the wide temporal range covered in these studies, often including a mix of acute, subacute, and chronic cases, which complicates the interpretation of outcomes due to the differing nature of these phases. Furthermore, the frequent crossover of patients from medical therapy to TEVAR indicates that many patients develop complications requiring intervention, which confounds the analysis and obscures the true effectiveness of the initial treatment strategies.

In response to the limitations of previous trials, there is a clear need for new studies with better patient enrollment and more precisely defined intervention timing. The Scandinavian Trial of Uncomplicated Aortic Dissection Therapy (SUNDAY Trial) is a significant step in this direction. This trial, involving 22 major aortic centers across five Nordic countries, aims to determine whether early TEVAR improves five-year survival in patients with uncomplicated TBAD. It plans to enroll 554 patients, randomizing them at least one week after symptom onset, with TEVAR performed within 90 days. This approach specifically targets the subacute phase, addressing the timing controversy [88].

The design of the SUNDAY Trial, with its pragmatic inclusion criteria and focus on local practices, aims to overcome the shortcomings of earlier studies. By focusing on a well-defined patient population and ensuring rigorous follow-up, this trial could provide crucial insights into the role of TEVAR in uncomplicated TBAD, helping to refine treatment protocols and improve patient outcomes.

These efforts highlight the ongoing need for well-designed trials from the outset, which are essential to generate robust, applicable evidence that can guide clinical practice and optimize patient care in TBAD management.

## 5. Imaging Support in Candidate Selection

Imaging plays a crucial role in selecting candidates for TEVAR in TBAD. Advanced imaging techniques facilitate diagnosis and provide detailed anatomical and functional information critical for decision-making.

CTA is the first-line imaging modality due to its rapid acquisition and high-resolution capabilities, allowing for detailed anatomical mapping of the aorta, identification of the entry tear, and assessment of the true lumen (TL) and FL. magnetic resonance imaging (MRI), particularly with 4D flow techniques, provides additional hemodynamic information that can predict complications and guide treatment strategies. positron emission tomography (PET) imaging, though less commonly used, can assess inflammatory activity in the aortic wall, potentially identifying patients at higher risk of disease progression.

Imaging is crucial in identifying radiological signs of impending rupture and malperfusion in aortic dissection, which are essential in guiding treatment decisions. In impending rupture, a CTA detects discontinuities in the aortic wall and periaortic hematomas with contrast leakage, while an MRI highlights acute hemorrhage with high signal intensity on T1 and T2 images. Rapid aortic expansion and hemorrhagic effusions are also key indicators. Malperfusion, resulting from dynamic or static obstruction of arterial branches, leads to organ ischemia. Both CTA and MRI reveal the intimal flap bowing into the TL or artery narrowing due to thrombus formation, alerting clinicians to potential complications. Identifying these signs helps in the timely application of emergency interventions and shapes the flowchart for aortic dissection management [89, 90].

The timing of follow-up imaging in TBAD and the exact intervals between scans is a crucial yet often debated aspect of care. The guidelines recommend imaging at admission, on day 7, at discharge and at 6 weeks post-discharge due to the elevated risk of instability in the early phase. In stable patients without clinical or radiological indicators of complications, our protocol suggests repeating follow-up CTA scans at 7 days, in accordance with the guidelines, balancing the need for close monitoring with the goal of minimizing unnecessary radiation exposure and patient burden [90].

Imaging plays a dual role in the management of TBAD, both before and after treatment, by facilitating precise planning and monitoring of therapeutic interventions Preprocedural imaging is essential for planning the intervention to assess the aortic anatomy, locating the primary intimal tear, and evaluating the extent of the dissection and involvement of branch vessels. This information is crucial for selecting appropriate endovascular devices, determining the optimal landing zones, and identifying potential challenges that may arise during the procedure​. Postprocedural imaging, on the other hand, is vital for monitoring the success of the repair and detecting any complications early. It is used to confirm the exclusion of the FL, evaluate aortic remodeling, and identify complications such as endoleaks, stent-graft migration, or new entry tears.

The status of FL patency is a critical factor in predicting aortic growth and the need for future interventions. A patent FL is completely filled with contrast throughout its length on imaging, while partial thrombosis is indicated by the presence of both flow and thrombus. Complete thrombosis occurs when there is no contrast within the FL. However, evaluating thrombosis based only on arterial phase images can lead to overestimating its extent. The SVS and the STS define positive aortic remodeling as the reduction of the FL or the expansion of the TL without an increase in the overall aortic size, or as a decrease in total aortic diameter regardless of lumen changes [5].

Regular follow-up imaging allows for the assessment of aortic stability and helps guide further interventions if necessary. The use of both pre- and postprocedural imaging is indispensable in ensuring the long-term success of TEVAR, as it provides a comprehensive evaluation of both the immediate and delayed effects of the intervention​. The integration of these imaging strategies enhances patient outcomes by facilitating timely and precise interventions tailored to the specific anatomical and pathological features of each case of TBAD [1, 91].

### 5.1 CTA

CTA remains the cornerstone landmark for diagnosing and managing TBAD due to its widespread availability, speed, and high-resolution images. CTA provides a comprehensive evaluation of the entire aorta, from the aortic arch to the femoral arteries, which is essential for assessing dissection extent and planning TEVAR. The CTA protocol includes a non-contrast phase, essential for detecting intramural hematomas and intimal calcifications, followed by contrast-enhanced scans. Optimal acquisition timing is critical to ensure proper visualization of the TL and FL, and to evaluate dynamic changes in flow patterns within the aorta. The arterial phase, typically acquired 20–30 seconds after contrast injection, is essential for identifying the intimal flap and distinguishing between the true and FLs. The delayed phase, usually captured 60–90 seconds after injection, helps to assess slow-flow areas and thrombosis within the FL, as well as any leakage of contrast material that may indicate rupture or endoleak post-intervention [1].

The integration of electrocardiographically (ECG) gated CTA enhances image quality, especially in the aortic root, by minimizing motion artifacts. This technique allows for precise measurements critical in selecting the appropriate stent size and configuration. Despite the concerns about radiation exposure, computed tomography (CT) remains indispensable for its diagnostic accuracy and utility in urgent settings [92, 93, 94].

CTA constitutes an essential tool in determining which patients with TBAD require urgent intervention due to complications such as malperfusion syndromes and aortic rupture. Early detection of these complications is vital for selecting appropriate candidates for treatment.

**Fig. 2.**
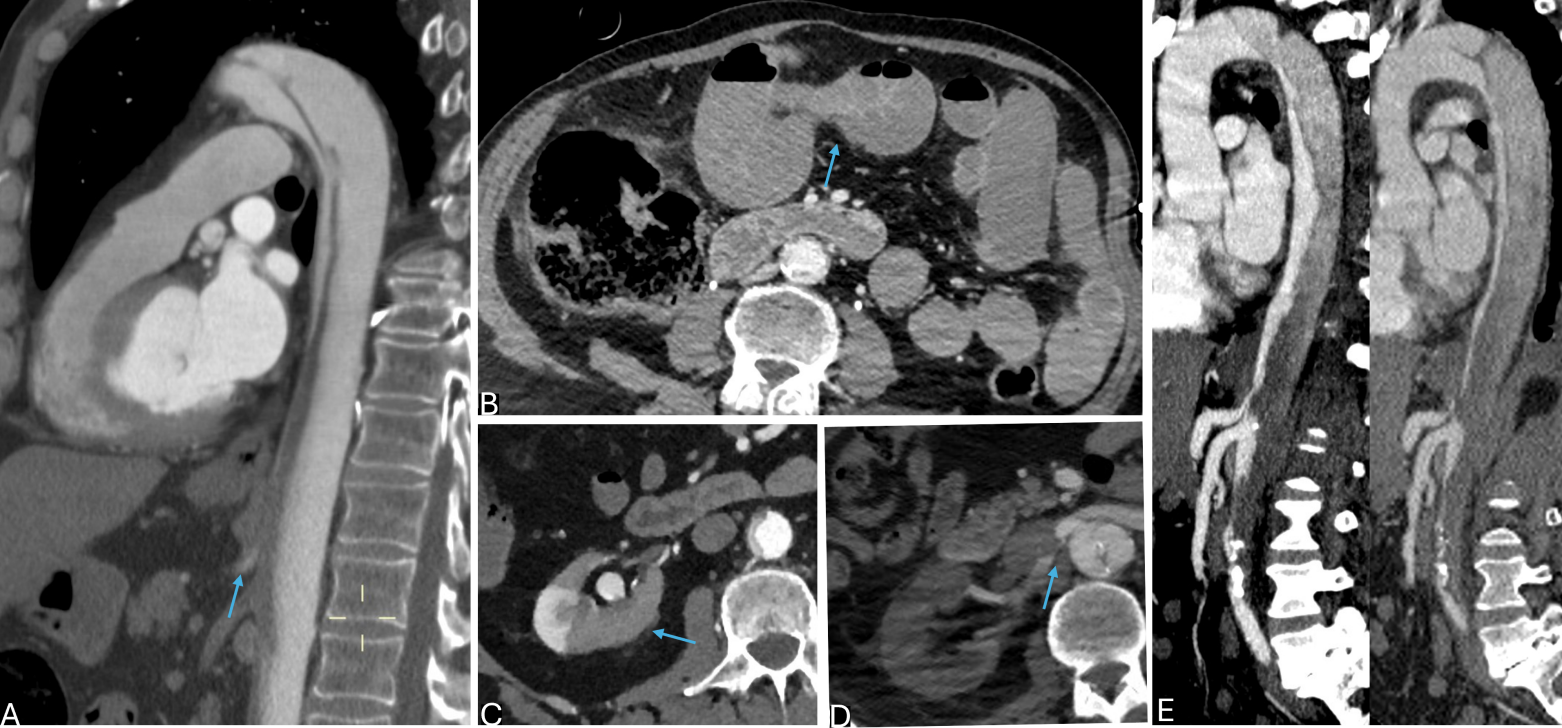
**Complicated type B aortic dissection (TBAD) at CTA: vascular and visceral malperfusion**. (A) Sagittal-reformatted computed tomography angiography (CTA) in arterial phase showing true lumen (TL) compression and occlusion of branch vessels (superior mesenteric artery shown, arrow). (B) Same patient, axial CTA in venous phase showing small bowel distension with fluid-air levels and scarce parietal enhancement (arrow) consistent with visceral malperfusion syndrome. (C) Axial CTA in the arterial phase showing intimal flap bowing towards the TL, with TL compression and associated right kidney ischemia (arrow). (D) Static malperfusion with narrowing of right renal artery due to focal false lumen (FL) thrombosis (arrow). (E) Dynamic malperfusion with diffuse compression of the TL.

Malperfusion syndromes occur when the FL created by the dissection obstructs blood flow to essential organs, leading to ischemia. In terms of CTA, this can be identified by several key features: the narrowing or occlusion of major aortic branch vessels, collapse of the TL, delayed contrast enhancement in affected organs and areas of hypodensity within tissues indicating ischemic damage (Fig. 2). These signs are crucial in identifying patients at risk of organ failure who may benefit from immediate intervention.

**Fig. 3.**
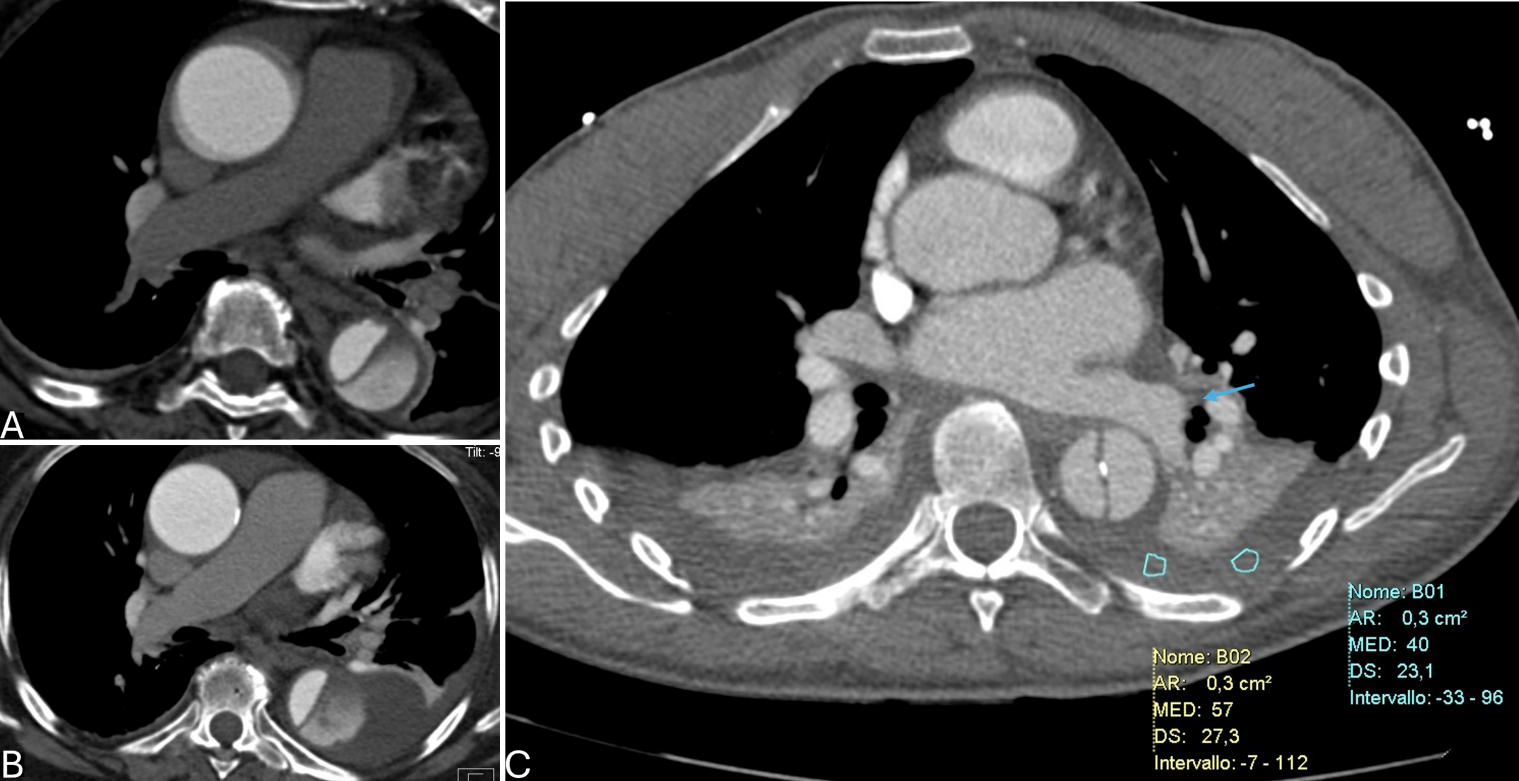
**Complicated type B aortic dissection (TBAD) at computed tomography angiography (CTA): impending rupture signs**. (A,B) Rapidly increasing pleural effusion at serial imaging at hospital admission (above) and 10-day follow-up CT (below). (C) Hemorragic pleural effusion (HU >30–40) and periaortic hematoma (arrow). HU, Hounsfield Unit; AR, area of the region of interest; MED, HU median value; CT, computed tomography; DS, standard deviation.

Impending aortic rupture is also readily detected by CTA. Key indicators include the presence of a periaortic hematoma, discontinuity of the aortic wall, hemothorax, active contrast extravasation and significant initial aneurysmal dilatation or any aortic diameter increase in the short time (Fig. 3). These findings suggest that the structural integrity of the aorta is compromised, warranting urgent endovascular repair [25, 95, 96].

Despite the importance of identifying these features, current clinical guidelines lack specific parameters for defining malperfusion, which introduces significant uncertainty in clinical decision-making. This is particularly challenging in situations where malperfusion is not clinically apparent. For instance, radiological signs of renal or mesenteric malperfusion observed on CTA may be asymptomatic, even without any increase of laboratory markers raising the question of whether these findings alone should prompt intervention. The dynamic nature of TBAD further complicates management, as patients initially classified as uncomplicated can rapidly deteriorate into complicated cases. Up to 40% of patients initially diagnosed with uncomplicated TBAD eventually develop complications underscoring the importance of regular surveillance and the potential benefit of early intervention [97].

In conclusion, while CTA is invaluable in identifying high-risk features of TBAD, the absence of specific guidelines for malperfusion complicates the selection of candidates for intervention. There is a pressing need for more precise definitions and evidence-based protocols to guide clinicians in identifying which patients are most likely to benefit from early treatment, particularly in the context of malperfusion and other high-risk features.

### 5.2 MRI

The use of MRI, particularly 4 dimension (4D)-flow MRI, has become an increasingly valuable tool in the assessment and management of TBAD. Traditional MRI provide limited insights into the hemodynamic forces at play within the TL and FL of the dissected aorta. This limitation has spurred interest in advanced imaging modalities like 4D flow MRI, which offers a comprehensive assessment of blood flow dynamics across the entire cardiac cycle.

4D flow enables the quantification of several hemodynamic parameters, including kinetic energy, peak velocity, and flow stasis within both the TL and FL. These parameters have been shown to correlate significantly with the risk of rapid aortic growth, defined as an increase in diameter of ≥3 mm/year. In particular, the kinetic energy ratio between the FL and TL was found to be positively correlated with aortic growth rates (r = 0.58, *p* = 0.01), highlighting the importance of dynamic pressure differences as a potential driver of disease progression [98, 99, 100].

Recent advancements in MRI technology, such as compressed sensing techniques, have reduced acquisition times and improved the feasibility of using 4D flow MRI in clinical practice. However, in the acute setting, MRI is not readily available which limits its use primarily to specific clinical scenarios, especially in settings where CTA might not be sufficient, during follow up, especially in young patients or in patients who cannot tolerate iodinated contrast media.

### 5.3 PET

PET, often combined with MRI, offers insights into the inflammatory processes associated with TBAD. Integrated 18F-fluorodeoxyglucose (FDG)-PET/MRI has demonstrated that the inflammatory activity in TBAD is most pronounced during the acute phase, particularly within the descending aorta. The highest FDG uptake was observed within two weeks of the dissection onset, with the target background ratio (TBR) reaching up to 5.8 in the descending aorta, indicating a hyperinflammatory state. This heightened inflammatory response was also detected, though to a lesser degree, in the ascending aorta and aortic arch.

Over time, the inflammatory activity measured by FDG uptake decreases significantly. By the subacute phase (three to four months post-onset), TBR values in the descending aorta dropped to approximately 3.5, and further decreased to around 2.9 by the early chronic phase (nine to twelve months). Interestingly, inflammation in the ascending aorta and aortic arch stabilizes earlier, typically within the first three to four months. These findings suggest a dynamic process of inflammation that could inform the timing of therapeutic interventions, such as TEVAR [101, 102, 103].

Although PET is not routinely used in all TBAD cases due to its limited availability and high cost, it shows significant promise for assessing the biological activity of aortic dissections and guiding personalized treatment strategies. Integrated PET/MRI systems, which offer enhanced evaluation of both structural and metabolic changes in the aortic wall may be reserved for complex cases where more detailed information is necessary beyond what CTA can provide.

## 6. Endovascular Treatment of Type B Aortic Dissection

**Fig. 4.**
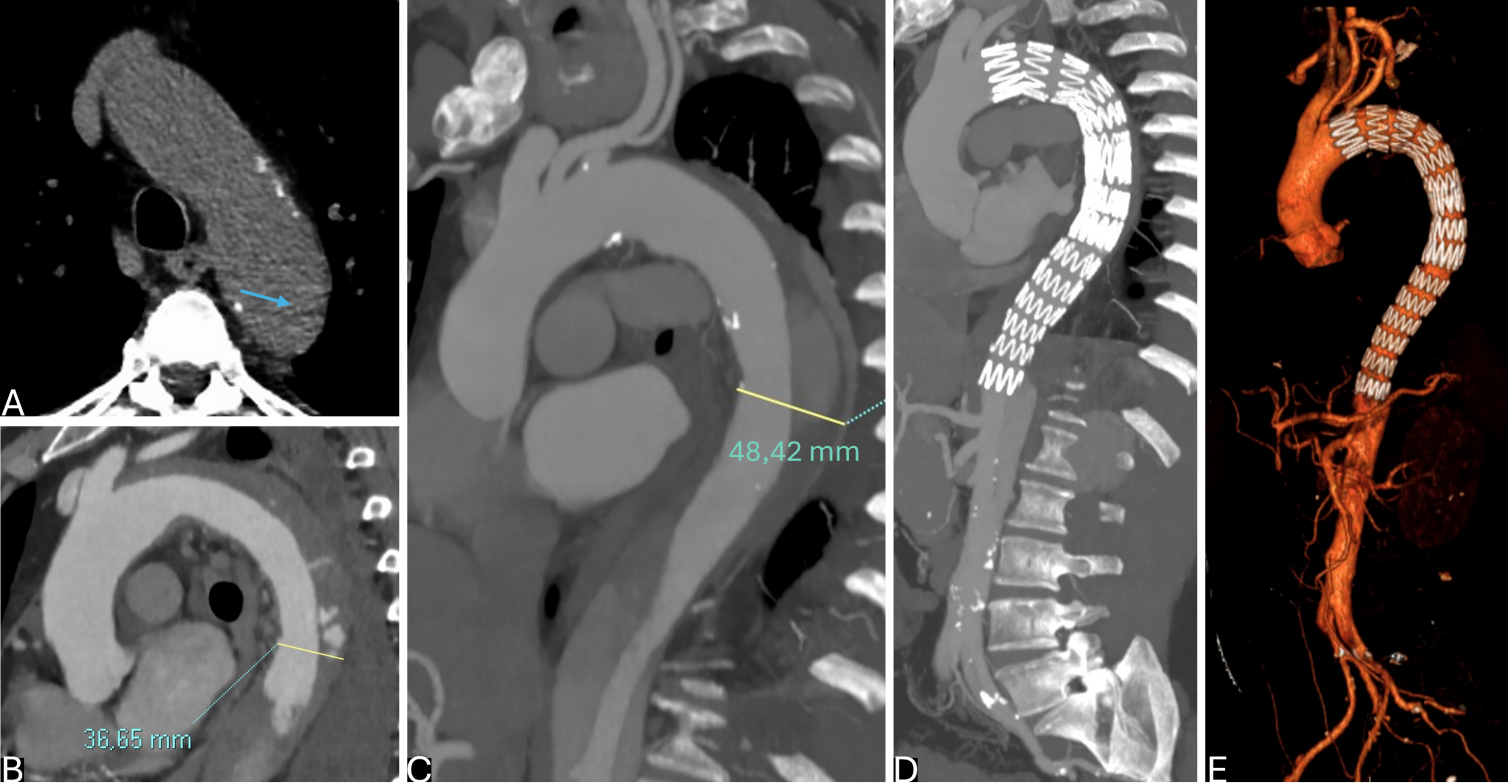
**Optimal timing of endovascular treatment in type B aortic dissection (TBAD) with rapid aortic growth during hospitalization and acute intramural hematoma (IMH) on distal arch**. (A) TBAD with retrograde IMH (arrow). (B,C) Sagittal-reformatted computed tomography angiography (CTA) at admission (B) and at 15 days (C) showing significant increase in aortic dimension with IMH reabsorption. Thoracic endovascular aortic repair (TEVAR) was performed in the subacute phase (1 month) due to high procedural risk. (D,E) Sagittal-Reformatted maximum intensity projection (MIP) CTA (D) and virtual reality (VR) CTA (E) at 3 months after treatment with almost complete false lumen (FL) shrinkage in the thoracic descending aorta.

Endovascular treatment has become a primary approach for managing TBAD due to its minimally invasive nature and the ability to improve patient outcomes while reducing recovery time and complications associated with open surgery. The primary objective of endovascular therapy is to exclude the FL, promote thrombosis within it, and stabilize or remodel the aortic wall. This is achieved using various stent-graft systems and adjunctive techniques tailored to address specific anatomical challenges and disease presentations (Fig. 4). The evolution of endovascular devices has led to the development of several key systems and techniques, each offering unique benefits for the management of TBAD [64, 104].

### 6.1 TEVAR Devices

5 thoracic TEVAR devices are approved by the U.S. Food and Drug Administration (FDA) for treating TBAD. These include the Zenith TX2 Dissection Endovascular System from Cook Medical Technologies (Bloomington, IN, USA), which comprises a proximal component known as the Zenith TX2 Dissection Endovascular Graft (ZDEG) with Pro-Form and a distal component, the Zenith Dissection Endovascular Stent (ZDES); the Valiant Captivia and Valiant Navion from Medtronic (Minneapolis, MN, USA); and the Conformable-TAG (CTAG) from W.L. Gore (Flagstaff, AZ, USA). All these devices are Conformite Europeenne (CE) marked for TBAD, and except for the ZDES, which is specifically designed for dissection, they also have FDA approval for other thoracic aortic conditions. Additionally, the Relay from Terumo Aortic (Sunrise, FL, USA) and the E-vita Thoracic 3G device from Cryolife Jotec (Hechingen, Germany) are CE marked for all thoracic aortic diseases, including dissection [105].

The ZDES, the first device specifically designed for TBAD, includes a proximal covered stent graft to seal the entry tear and a distal bare metal stent to support the TL and promote aortic remodeling. The ZDEG with Pro-Form, which can be used alone, differs from the Zenith TX2 graft by not having a proximal barb crown to prevent migration. Constructed from woven polyester and self-expanding stainless steel Z stents, the ZDEG improves wall contact for better sealing and reduced damage. The Pro-Form system allows for controlled deployment despite lacking a free-flow configuration. Two prospective multicenter studies sponsored by Cook evaluated the combination of ZDEG and ZDES (Stable I and II). These studies confirmed the approach’s feasibility, reporting a mortality rate of 5.5% and 6.8% respectively, for acute complicated TBAD. However, a limitation of the ZDEG with Pro-Form is the use of stainless steel in the stents, which can cause artifacts in MRI and prevent significant reduction of the system’s profile [43, 106, 107].

Medtronic’s Navion and Valiant stent-graft systems are engineered for flexibility and ease of deployment in the thoracic aorta. The Navion stent graft offers a low-profile delivery system that enhances manoeuvrability and precision during placement. The Valiant graft, known for its wide range of sizes and configurations, provides a custom fit for diverse aortic anatomies, ensuring effective exclusion of the FL. Both systems are made from self-expanding nitinol stents covered with durable polyester fabric. The Medtronic Dissection Trial, a non-randomized study, demonstrated the device’s safety and effectiveness for acute complicated TBAD, with a 30-day mortality rate of 8% and 15% at 12 months [108].

The Gore TAG® and CTAG® thoracic stent grafts are widely used in the endovascular repair of TBAD. These devices feature a flexible and conformable design that allows them to adapt to the aortic anatomy, providing effective sealing and support. The TAG stent graft is constructed from expanded polytetrafluoroethylene (ePTFE), which is durable and biocompatible, reducing the risk of endoleaks and promoting long-term stability. The CTAG further enhances this adaptability, making it suitable for a range of aortic arch anatomies. In a prospective multicenter study conducted by Cambria *et al*. [109], involving 50 patients who underwent TEVAR for complicated TBAD, the results were favorable, showing an in-hospital mortality rate of only 8% and promising outcomes 1 year after discharge.

The Relay device from Terumo Aortic (Sunrise, FL, USA) consists of a self-expanding nitinol frame combined with a polyester graft. It comes in two configurations: Relay Plus and Relay NBS Plus. The main difference is the presence of a proximal bare stent in the free-flow configuration, which enhances stability and reduces migration risk, but can potentially harm fragile aortic walls during dissection. In the NBS version, the support stents are sewn into the fabric, reducing the risk of iatrogenic dissection. Proximal sealing is achieved by coupling two support stents, allowing stable deployment. A retrospective study involving 78 patients, including 35 with TBAD, showed precise stent-graft deployment with no type Ia endoleaks and 82% accuracy in achieving less than 5 mm from target vessels post-operatively [110]. Although the Relay NBS lacks specific approval for aortic dissection, it functions effectively as a dedicated endograft for such cases. Both configurations offer a wide range of sizes, including 4 mm tapered prostheses, which are currently custom-made to address the size mismatch between proximal and distal aortic segments.

The RESTORE trial, a multicenter clinical registry, demonstrated the Relay device’s safety and efficacy across various thoracic aortic pathologies, including 96 TBAD cases, showing a 78.5% survival rate at 2 years, even with angulated aortic anatomies [79, 110].

The E-Vita Thoracic graft from Cryolife Jotec (Hechingen, Germany) consists of self-expanding nitinol stents and polyester fabric, available with either a bare stent or twin stent design for enhanced radial force. Although it offers ultra tapered versions with significant diameter differences, it lacks a configuration specifically for TBAD. The newer E-nya device, approved in Europe, is designed specifically for TBAD, featuring stents sewn inside the graft for uniform radial force distribution and a multifilament fabric for better conformability. It also includes a more tapered tip for easier delivery. A post-market study will assess its effectiveness in TBAD treatment.

### 6.2 Technique and Other Devices

**Fig. 5.**
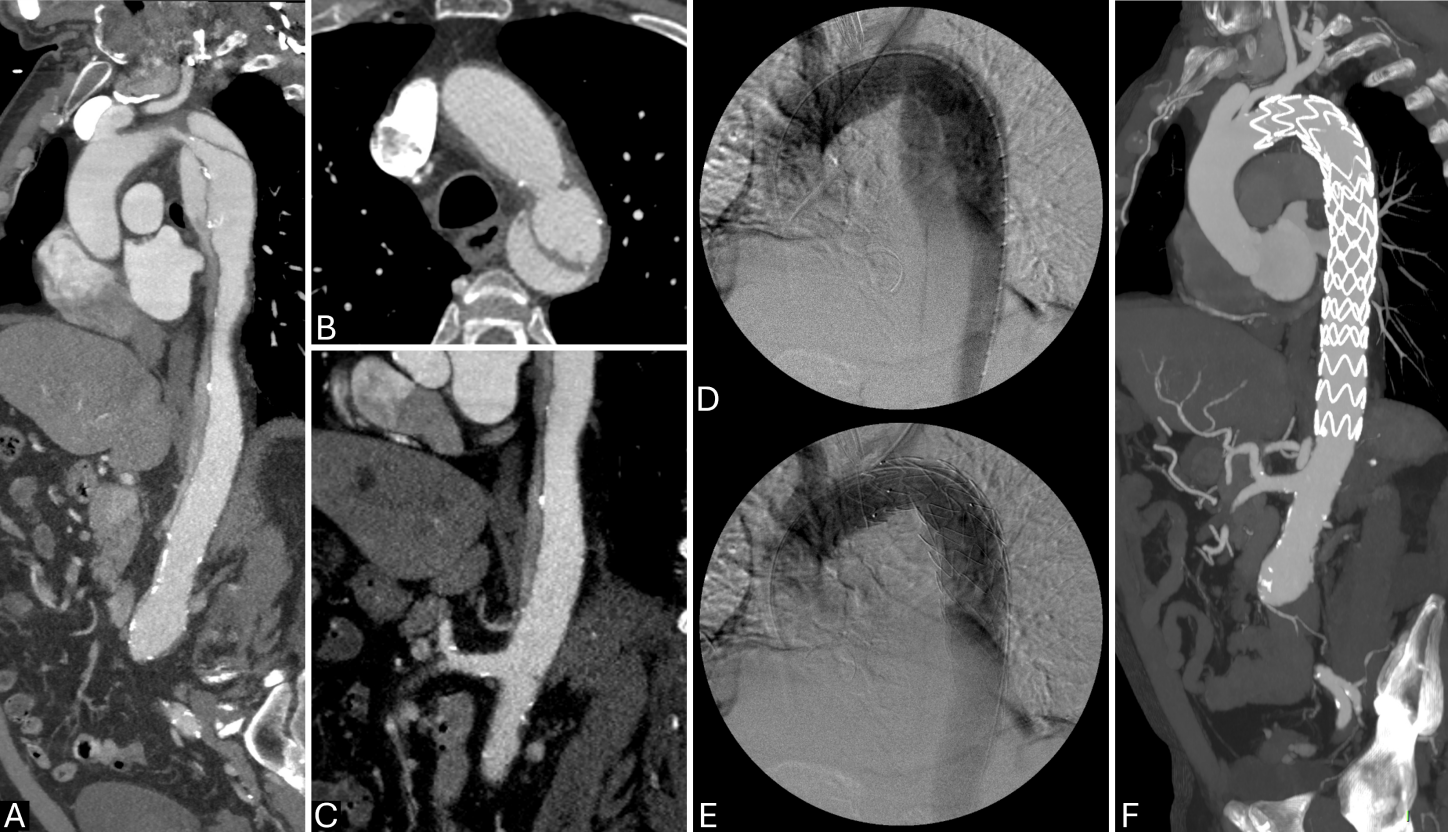
**Endovascular techniques for complicated type B aortic dissection (TBAD): PETTICOAT approach**. (A–C) Sagittal and axial computed tomography angiography (CTA) showing complicated TBAD with static malperfusion (due to false lumen (FL)-celiac trunk thrombosis and rising creatinine serum level due to intimal flap extending in left renal artery). (D,E) Digital subtraction angiography (DSA) showing thoracic endovascular aortic repair (TEVAR) plus bare stent implanted in abdominal aorta to expand true lumen (TL). (F) Sagittal-reformatted CTA at 7-year follow-up showing long-standing positive aortic remodeling expansion of the TL and no aneurysmatic evolution of the abdominal aorta.

A persistent issue in TBAD treatment is the unpredictable remodeling of the distal abdominal aorta, even after successful thoracic stent graft placement. Larger distal re-entry points often prevent thrombosis and remodeling of the thoraco-abdominal FL, potentially leading to complications like thoracic FL aneurysms, which may necessitate further interventions. To address this, Nienaber *et al*. [111] proposed the PETTICOAT technique (Fig. 5), involving closure of the primary entry tear with a standard stent graft, followed by deploying bare stents distally to encourage intimal flap attachment to the aortic wall. This method aims to expand the TL and encourage FL thrombosis without compromising distal perfusion. The bare metal stents provide radial force to support the TL, while the stent graft excludes the primary entry tear. This combination improves the hemodynamic environment within the aorta, promoting healing and reducing the risk of complications. The STABLE I study reported favorable five-year outcomes with PETTICOAT, demonstrating improved thrombosis and stability of the FL, particularly in the abdominal aorta, for both acute and chronic patients [80, 112].

**Fig. 6.**
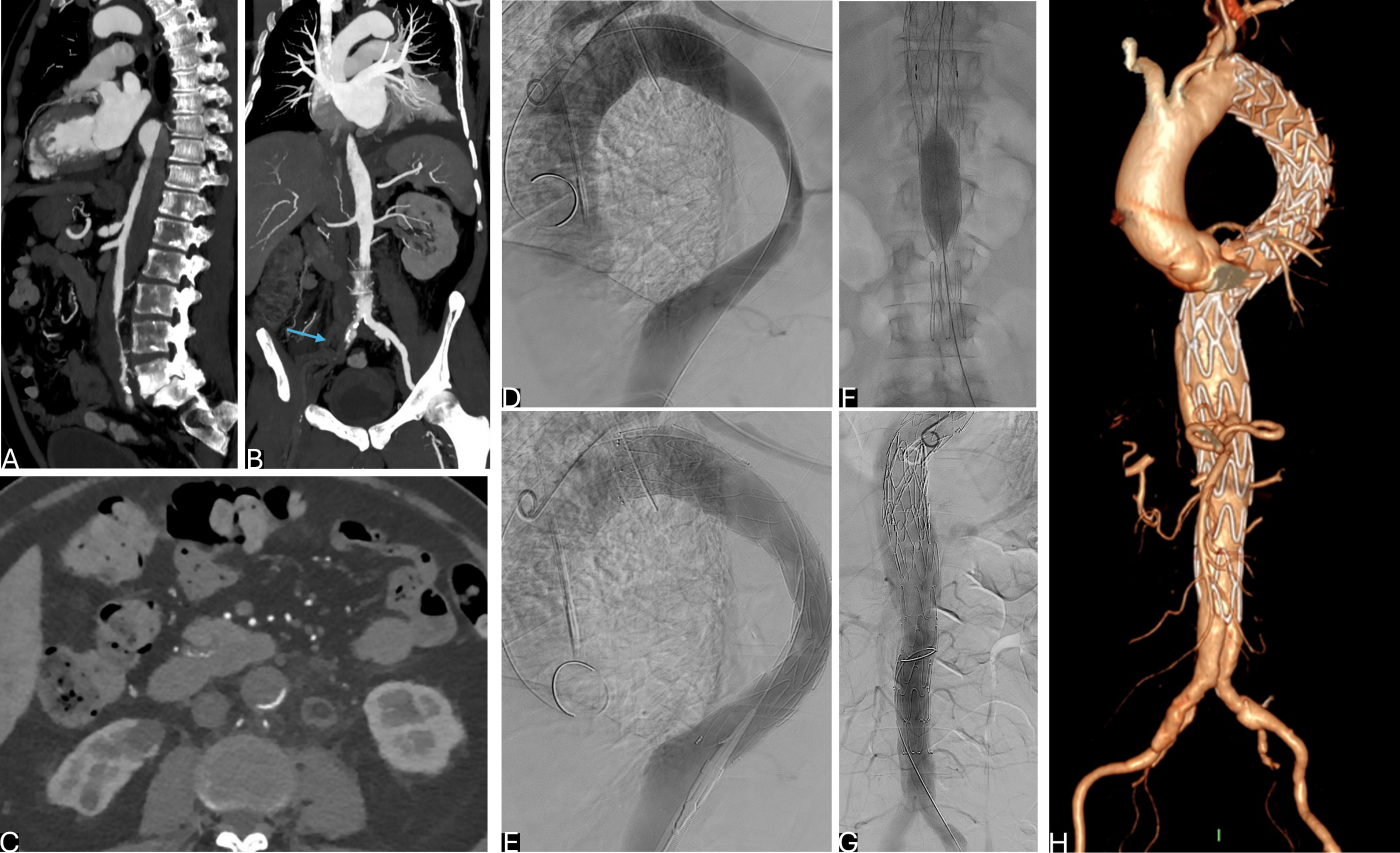
**Endovascular techniques for complicated type B aortic dissection (TBAD): STABILIZE approach**. (A–C) Sagittal, coronal and axial computed tomography angiography (CTA) showing TBAD with severe true lumen (TL) compression and right ilio-femoral artery occlusion (arrow) complicated by right limb ischemia. (D) Intraoperative digital subtraction angiography (DSA) showing entry tear in thoracic aorta. (E) DSA after stent graft deployment. (F) Intrastent ballooning in abdominal aorta to stretch the intimal flap. (G) Final DSA after thoracic endovascular aortic repair (TEVAR). (H) Aortic virtual reality (VR) at 1-year follow-up.

Hofferberth *et al*. [113] advanced the PETTICOAT technique with the development of the STABILISE method. This approach involves using a covered endograft in the proximal descending aorta, followed by a bare metal stent for distal aortic relamination, and then performing balloon-induced intimal disruption and reapposition (Fig. 6). However, using a molding balloon in areas with a bare metal stent is not recommended in the instructions for use. While initial results from single-center studies were promising, more research is needed to confirm the long-term safety and effectiveness of STABILISE, as it hasn’t been widely adopted due to concerns about potential aortic rupture during ballooning. Melissano *et al*. [114] introduced a modified version of the STABILISE technique with favorable early results, which refined several key aspects, including the maximum aortic diameter (TL and FL) for treatment, the choice of bare stent diameter, the type of balloon used, and the catheterization of branches from the FL. After completing the STABILISE procedure, a bare or covered stent can be deployed to connect the aortic TL with the target vessel [113, 114].

The PETTICOAT approach has led to the creation of specialized aortic bare metal stents, such as the ZDES and E-XL® aortic stent, designed to improve aortic remodeling in the treatment of TBAD. The ZDES, developed by William Cook Europe, is a single cylindrical device made from nitinol stents available in multiple lengths and two diameters (36 and 46 mm). It is designed to exert a gentle radial force to promote positive aortic remodeling without risking intimal flap rupture. Its wide mesh configuration facilitates stenting of visceral vessels post-deployment. The E-XL stent from Cryolife Jotec is a nitinol, self-expanding bare stent approved for TBAD and other aortic lesions. It comes in a range of sizes and features a closed-cell design for stability. The E-XL’s higher radial force is beneficial for static malperfusion cases, and studies like the ASSIST trial have shown excellent aortic remodeling outcomes with minimal risk of late complications [115].

Various alternative approaches have been developed to achieve complete FL thrombosis and favourable aortic remodeling in TBAD, using a range of embolization devices. These techniques can yield optimal results, particularly in emergency situations. One such method is the Candy-Plug technique, which involves deploying a modified endograft into the FL. This graft has a middle section occluded by an Amplatzer Vascular Plug after release. A thoracic endograft is placed in the TL above the celiac trunk, and proper alignment with the candy-plug is crucial to prevent stent-induced new entry (SINE).

The Knickerbocker technique uses a device with a double-tapered configuration and a larger central section. After deployment in the TL, the central section is ballooned to rupture the septum and extend the graft to the FL wall. Both techniques aim to occlude the FL proximal to the reno-visceral vessels, preventing backflow into the enlarged thoracic FL [116, 117, 118, 119].

## 7. Future Directions

Artificial intelligence (AI) is playing an increasingly pivotal role in modern medicine, revolutionizing how healthcare is delivered and managed. AI technologies are being utilized to enhance diagnostic accuracy, streamline administrative tasks, and personalize treatment plans. Machine learning algorithms, for instance, can analyze vast amounts of medical data to identify patterns and predict disease outcomes, aiding in early detection and decision-making processes [120, 121].

In the context of TBAD, AI algorithms, particularly those utilizing deep learning techniques such as convolutional neural networks, are being deployed to automate the detection and quantification of aortic dissections in CTA. AI is increasingly recognized as a transformative tool in the management of TBAD. AI algorithms have the potential to enhance diagnostic accuracy, improve treatment outcomes, and streamline patient management processes [122].

AI algorithms, particularly those utilizing deep learning techniques like convolutional neural networks (CNNs), are being increasingly used to automate the detection and quantification of aortic dissections in CTA. These algorithms significantly reduce the likelihood of missed diagnoses and improve inter-reader agreement, making them invaluable tools for radiologists. For instance, AI-based methods have shown high accuracy, sensitivity, and specificity in identifying aortic dissection, thus supporting clinical decision-making by providing precise measurements of the aortic diameter [123].

One notable application of AI in TBAD management is the development of predictive models that integrate radiomic features with clinical biomarkers to forecast postoperative complications. These models utilize machine learning techniques like CNNs to analyze CTA images, extracting detailed information that aids in the early identification of patients at higher risk of adverse outcomes. This approach not only improves diagnostic accuracy but also supports the selection of suitable candidates for TEVAR, ultimately enhancing patient outcomes.

Beyond diagnostics, AI is also playing a pivotal role in patient selection for TEVAR. Machine learning models have been developed to predict patient outcomes based on various clinical and radiological parameters, enabling more personalized treatment approaches. Studies have explored the use of AI to assess sarcopenia—a condition characterized by loss of muscle mass—which has been linked to poorer outcomes in patients undergoing TEVAR. By integrating sarcopenia assessment into preoperative evaluation, AI helps identify patients who might benefit from targeted interventions, such as nutritional support or physical therapy, before undergoing surgery [124].

Overall, the incorporation of AI in TBAD management represents a significant step forward in the field, offering tools that improve the precision and personalization of care. As AI technologies continue to evolve, their application in TBAD promises to enhance patient outcomes and optimize healthcare resources.

## 8. Conclusions

The management of TBAD stands at a pivotal crossroads, where emerging evidence and technological advancements are reshaping established practices. While CTA remains the undisputed cornerstone for diagnosis and monitoring, its true potential lies in the early detection of high-risk features, guiding clinicians toward timely, life-saving interventions. However, imaging alone is not enough, and so imaging data should be embedded within accurate interpretation of clinical signs and swift decision-making, which are critical to prevent severe complications.

TEVAR has revolutionized the treatment landscape for complicated TBAD, but its application in uncomplicated cases remains a subject of ongoing debate. As evidence begins to suggest that preemptive TEVAR may reduce long-term complications [19, 87], the challenge lies in identifying the right patients at the right time. Unfortunately, the lack of high-quality data from large randomized clinical trials limits the ability to make strong recommendations for routine TEVAR in uncomplicated TBAD to prevent long-term aortic-related complications. This underscores the pressing need for personalized, patient-centered strategies that move beyond rigid treatment algorithms, embracing the complexity and dynamic nature of TBAD, even throughout more challenging imaging technologies.

The moment calls for a more nuanced, evidence-based framework that balances risk with the potential for proactive intervention. This review emphasizes the importance of confronting ongoing controversies, addressing gaps in the literature through well-designed studies and innovative solutions. The future of TBAD treatment depends on refining tools, enhancing strategies and making bold, patient-centered decisions, that prioritize outcomes over conventional approaches.
